# Computational Epidemiology

**DOI:** 10.1109/MCSE.2010.7

**Published:** 2010-01

**Authors:** Pam Frost Gorder

## Abstract

Born from a desire to predict the future, epidemiology has largely been limited to studying the past. Now, computational epidemiology researchers are harnessing computing power to crack the complicated mystery of how diseases spread. Born from a desire to predict the future, epidemiology has largely been limited to studying the past. Now, computational epidemiology researchers are harnessing computing power to begin to crack the complicated mystery of how diseases spread.

When news reports declared that two well-publicized computer models underestimated the initial spread of the 2009 swine flu pandemic, people asked why the models didn't work better. But the real question is why the models worked as well as they did given the difficulty that scientists face in tracing the human behavior patterns that spread disease.

### The Prediction Challenge

As Armin Mikler, director of the Computational Epidemiology Research Laboratory at the University of North Texas, explains it, the science of epidemiology sprang from the human desire to predict the future. Ever since 19th century doctor John Snow traced a deadly outbreak of cholera to certain London water wells, scientists have attempted to track human behavior to forecast—and curtail—the spread of disease. From those roots, epidemiology has grown into a broad discipline.

Mikler, like many epidemiologists around the world, works with doctors, statisticians, social scientists, computer scientists, and public health officials to sort through the myriad genetic and environmental factors that promote disease. In the US, critical data comes from the Centers for Disease Control and Prevention (CDC). The goal is to one day track every illness—from cancer and heart disease to obesity and alcoholism. Epidemiologists have their work cut out for them: given the rate of international travel today, any communicable illness has the potential to cross the globe in a matter of hours.

Traditionally, researchers have examined past outbreaks, working backward to pinpoint likely causes. As a result, Mikler says, the science of public health "has become very good at analyzing what has happened, but is not very well equipped to predict what might happen." As Carlos Castillo-Chavez, director of the Mathematical, Computational, and Modeling Sciences Center at Arizona State University, puts it: "We can't do experiments. We can't infect someone and see what happens. We have to make decisions based on limited data."

By analyzing past outbreaks, epidemiologists are working to pinpoint factors that will most influence outbreaks in the future. Such predictions are difficult, however, because human behavior is notoriously random. When people are sick, they might go out or stay home. They might see a doctor or not. And, if they do visit a doctor, that doctor might run tests or simply diagnose the problem using his or her own best judgment. All such behaviors are essentially invisible to scientists and clearly complicate the prediction task.

Still, as Castillo-Chavez notes, there's tremendous public pressure to generate specific predictions, such as the number of people who will become infected. "We demand to know—even though science has shown that prediction is rarely a possibility."

### A Model Case: Swine Flu

With the availability of massive data storage and fast processors, computational epidemiology has developed in the hope of filling the knowledge gap by simulating the spread of disease. Using computers to find patterns in data can help guide public health policy decisions, including how to distribute limited resources such as vaccines.

Swine flu efforts in the US offer a recent case in point and also illustrate the challenges facing the still-nascent field of computational epidemiology. One of the most prominent 2009 swine flu computer models came from Dirk Brockmann, professor of engineering and applied mathematics, and his team at Northwestern University. Their model correctly pegged the disease as entering the US from Mexico, with the most intense outbreaks in California, Texas, Florida, and New York.

In its first projection on 3 May 2009, the model estimated that by that month's end there would be approximately 2,000 cases in the US—a number Brockmann describes as having "an enormous error bar." This initial number was widely reported in the press. When, at the end of May,

1521-9615/10/$26.00 © 2010 IEEE

the CDC reported 7,500 confirmed cases—and an estimated 100,000 unreported cases—the *The New York Times* ran a story that asked, "What went wrong?"[1]

In fact, nothing had gone wrong. Brockmann's team had continued to refine the simulations, and by 5 May their estimate was that approximately 7,000 cases would occur by 17 May—a projection that would raise the possible number of cases to 100,000 by month's end. So, after three simulation trials, the team was actually pretty close to the CDC's own report on the number of potential cases. (As of early September, the number of confirmed US cases was just under 44,000, with 302 people dead, and an estimated 1 million cases unreported; numbers have since continued to rise dramatically.)

The model's initial numbers were low because the team had underestimated the number of initial infections in Mexico. Once corrected, the projections fell in line with CDC estimates. Brockmann's success suggests that computer models can effectively help guide public policy—when good initial data is available, that is. But where do those initial numbers come from? Ultimately, they're based on suggestions from public health officials and knowledge about human behavior.

## The Social Network Model

Epidemiology has grown more mathematical over the past century, according to Madhav Marathe, a professor of computer science and the deputy director of the Network Dynamics and Simulation Science Laboratory at Virginia Tech's Virginia Bio-Informatics Institute.

Marathe and Keith Bisset, a senior research associate at the NDSS Lab, note that disease models based on simple differential equations and aggregate data worked well before the Earth's population became urbanized and mobile. Now diseases thrive in crowded cities and are easily carried abroad, creating large, complex social networks.

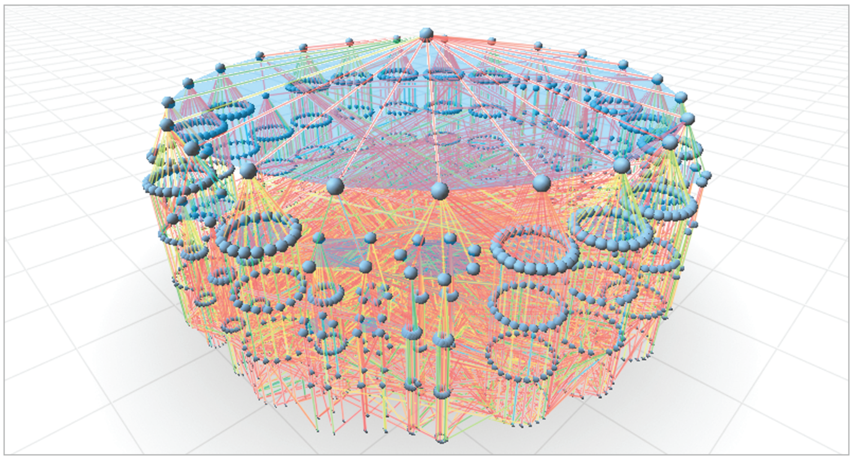

Figure 1. A computational epidemiology model that simulates social contact in a large population. This model, used at Virginia Tech, shows a slice of the complete social network for a "typical" person living in a simulated city based on Chicago. (Image courtesy of David R. Nadeau, San Diego Supercomputer Center.)

To contend with this complexity, researchers have begun to base their epidemiological models on computational networks—mathematical constructs of real-world networks. As Bisset points out, many of the basic principles of network theory that apply to particle physics, transportation science, and economics also apply to epidemiology. That's because network theory describes complex interactions and relationships between generic objects, or network nodes. Individual nodes interact with other nodes based on network connections; in social networks, individual attributes—including a person's behavior and interactions with others—determine the course of a disease over time (see Figure 1).

## Consensus Building

Like the other researchers interviewed for this article, Brockmann and his team run their network-based models on computer clusters with multicore processors. "Every simulation we run is different, because we also simulate random events," Brockmann says. "In order to get good statistics, we may run 1,000 pandemic events and then compute the expected outcome by averaging. We want to be able to adjust our simulations on a daily basis during the initial outbreak of an epidemic. Therefore, we need very fast computers, and lots of them."

Brockmann's team starts with small clusters for coarse-grained simulations, and then moves on to larger clusters for more detail; they ran their most detailed swine flu model on the BlueGene supercomputer at Argonne National Laboratory. According to Brockmann, a model is ready for public consumption when it's structurally stable. "That means, when you slightly alter the equations that are involved, the qualitative features of the model dynamics do not change," he says, adding that "you have to have a good understanding of how the various dynamical ingredients interact individually before you add them all together in a complete model."

Thus, to ensure that their assumptions are valid, computational epidemiologists must work closely with statisticians and public health experts. As Marathe notes, "consensus building is very important." It's also important to set a context before releasing results, according to Castillo-Chavez, who says that emphasizing all the caveats is crucial before unveiling a model

## Observatoire Landau

### Discoveries Arising from Computing

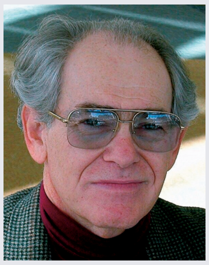

*By Rubin Landau, Department Editor*

Fleeting, off-the-cuff remarks by colleagues, parents, and spouses somehow have the ability to stick with you for unreasonably long times. I recall remarks by colleagues to the effect that, "if you were any good as a theorist, you would not need to do computing," and that what we need are "pencil and paper theorists who think about things." Although I believe that many of these types of remarks (already given too much attention in an earlier sidebar), are just examples of self aggrandizement, they've probably led me to prepare a defense by pondering the question, "What important scientific discoveries would not have been possible without computing?" Particularly of interest to me are the original, creative, and beautiful developments that make science so interesting. Even though I've now given up trying to make those discoveries myself, the question still interests me; here, I present some thoughts I gathered for a talk I gave at a recent Gordon Research Conference.

Some of the first examples I became aware of come from what we now call *nonlinear science*, a field in which many of the discoveries were made computationally and then cleaned up and derived by mathematicians. For example, while the discovery of solitons probably should be credited to John Scott Russell's 1834 observations and calculations, I believe it was the numerical studies of Enrico Fermi, Stanislav Ulam, and John Pasta in 1955 and Norman Zabusky and Martin Kruskal in 1965 that led to the field's blooming. Likewise, while Henri Poincaré studied chaos in the 1880s, it seems to me that it was the numerical studies of Edward Lorenz in 1961 that led to the modern progress in the subject.

Probably my most basic example is the field of lattice quantum chromodynamics, in which computation is helping to prove that QCD is not only the first real theory for strong interactions, but also a viable one. Here, I believe Ken Wilson deserves the credit for realizing early on that solutions to these complicated and highly nonlinear field equations were possible only via Monte Carlo simulations. Recent times has seen continuing improvement in the predictions due to increasing computation power, improved theory, and improved algorithms all developing hand in hand.

While speaking of particle physics, let us not forget the critical place computation and simulation have in particle experiments. Indeed, many of the major experiments at Fermi Lab, CERN, and the Large Hadron Collider are sophisticated and subtle mixes of observation, simulation, reconstruction, and analysis that have changed what we mean by "seeing" a particle, as well as changed the way other sciences are now done. (Need I remind you that the World Wide Web originated at CERN to support these collaborations, with their huge quantities of multimedia data that had to be handled by scientists the world over? But that turns the question around into major developments in computing arising from the need to do science, something that nuclear and particle physics have been doing for quite some time.)

One of the recent advances in science that I find most interesting is the integration of the data-intensive to an impatient public. "We have to be clear about our assumptions … there should be truth in advertising."

In an effort to build better models and thus produce more reliable results, researchers are digging up data in innovative ways. Brockmann's team, for example, used data from Where's George?—an Internet site that tracks the movements of dollar bills—as a proxy for face-to-face human contact. Mikler's team chose a different proxy: blog postings. Hoping that bloggers who caught the flu would write about it, they downloaded some 10 Tbytes of blog entries between October 2008 and August 2009. So far, they're finding a relatively strong correlation between blogs and CDC data.

### Looking Forward

With better data sources, Marathe believes that computational epidemiology could soon become less of a predictor and more of a real-time tracking tool. He foresees more work being done on supercomputers, with increasingly elaborate simulations produced rapidly, as an epidemic unfolds.

Initial efforts toward this goal are already under way. At the University of North Texas, Mikler's team has built a simulation chamber—a kind of "situation room"—in which computer scientists, epidemiologists, and public health officials can gather to visualize disease data from multiple sources on a large screen, manipulate the data, and make real-time decisions. At Arizona State, Castillo-Chavez oversees a similar laboratory, the Decision Theater (www.decisiontheater.org), which enables real-time surveillance in a dynamic, visual way.

Martin Meltzer, senior health economist at the CDC, notes that a key challenge will be for researchers to show all this elaborate data in a way that's simple to understand, but not so simple that important information is lost. Also, because the CDC must issue recommendations to public health officials, all models must be easily accessed on desktop computers. "I spend my time building models that people can download from the 'Net," says Meltzer.

That's precisely why Bisset, Marathe, and their colleagues at Virginia computational tools (and people) of particle physics with the Sloan Digital Sky Surveys and the digital tools of astronomy. This, when combined with multiscale and multiphysics simulations (discussed next), seem to have turned what used to be an observational science into an experimental one. As an example, consider the supernova-on-demand developments at Lawrence Berkeley Lab. Supernova are very much the standard candle of astronomy and have permitted us to measure the expansion rate of the universe and thereby infer information about the amount of dark energy it contains. Here, scientists use computations with a two-point correlation function over tremendously large data sets to find changes in temporally separated images of selected regions of the sky, and thereby deduce the presence of type 1A supernova. Amazingly, a dozen supernovas have been found while still brightening.

Another example of applying particle physics computation in astronomy is the Amanda Neutrino Experiment. It employs a detector array for its Cerenkov counter that is three times the size of the Eiffel Tower and is buried a mile deep in the ice of Antarctica (the ice is the light source). The volume of data produced is large (15 Tbytes/year), with the data stored and analyzed using the TeraGrid. This experiment has produced a picture of the very high energy neutrino sky, which remains a mystery.

As just hinted at, astronomy simulations have also led to major scientific discoveries. These simulations are fundamentally different from those of QCD in which one solves the equations provided by a single physical description. Many simulations, such as those of galaxy and star formation or complex materials, are multiscale and multiphysics models in which the same equations are solved at widely different scales and then (somehow) matched together at the interfaces. We can think of these simulations as hybrid calculations that combine discrete and continuous models, use adaptive multiscale grids, and apply stochastic and deterministic algorithms. As you might imagine, it's often quite hard to put the disparate pieces together ("what God has joined together let no man put asunder").

While speaking of astronomy, I'd be amiss not to mention the simulations and animations of the collision of two black holes. The calculations are challenging and intensive, and predict a Jell-O-like shivering of space-time that leads to gravitational waves throughout the universe. Observing these gravitational waves is still an unfound holy grail.

Although I fear I tread on thin ice when discussing biology, my foundation is reinforced by using an example cited by the Pittsburgh Supercomputing Center's Ralph Roskies. In a talk, Roskies discussed how the 1993 Noble Prize in chemistry was given to Peter Agre for his advances in understanding how aquaporins transmit water, but not other molecules, in both directions through cell walls. Not only did Agre's work employ extensive molecular dynamic simulations to arrive at that understanding, it also produced an animation of the process—an animation mentioned by the Noble Prize committee (an historical first).

Finally, let me end by noting that the collection of codes and data known as the "cosmic simulator" has shown how the scientific ideas first put together in Steven Weinberg's *First Three Minutes* form a robust base for computing the formation of galaxies and the modern universe from the Big Bang. I call that important. I'd be thankful to learn about your examples.

Tech developed Simdemics software, which lets officials with different levels of computational experience set up detailed experiments to study various "what if" scenarios. Simdemics has three variants: EpiSims, EpiSimdemics, and EpiFast, which let users trade off between model generality and processing speed.

Ways to model the human aspect of disease dissemination will continue to evolve. In the future, Brockmann believes that models will simulate not just the spread of a disease but also people's fear of it, which changes their behavior and thus alters the disease's course. "Despite the enormous detail many models have nowadays, this sort of feedback loop has not been investigated systematically yet," he says. "Based on new Internet technologies, I believe this could be accomplished."

Bisset agrees. Modeling people's behavioral responses to an epidemic is "a beautiful question to tackle in the next few years," he says. He and Marathe have added a behavioral feedback loop to Simdemics, and are now testing it. As Brockmann cautions, however, increases in model complexity and computing power won't automatically translate into greater understanding of diseases. "I think we need to unravel the underlying structures that shape the patterns and dynamics of infectious diseases," he says. That notion meshes with Meltzer's general message from the CDC: keep it simple. "I would issue this challenge: can you reduce your model to a spreadsheet? Then you have a chance of connecting with policy makers."
